# Arrhythmias and Conduction Disturbances in Patients with Systemic Sclerosis—A Systematic Literature Review

**DOI:** 10.3390/ijms232112963

**Published:** 2022-10-26

**Authors:** Cristina Andreea Vrancianu, Ana Maria Gheorghiu, Dragos Emanuel Popa, Jeffrey Shi Kai Chan, Danish Iltaf Satti, Yan Hiu Athena Lee, Jeremy Man Ho Hui, Gary Tse, Ioan Ancuta, Ana Ciobanu, Mihai Bojinca

**Affiliations:** 1 Faculty of Medicine, Carol Davila University of Medicine and Pharmacy, 050474 Bucharest, Romania; 2 Internal Medicine and Rheumatology Department, Cantacuzino Hospital, 020475 Bucharest, Romania; 3 Cardiology Department, Theodor Burghele Hospital, 050653 Bucharest, Romania; 4 Epidemiology Research Unit, Cardiovascular Analytics Group, China-UK Collaboration, Hong Kong; 5 Kent and Medway Medical School, Canterbury CT2 7FS, Kent, UK; 6 Tianjin Key Laboratory of Ionic-Molecular Function of Cardiovascular Disease, Department of Cardiology, Tianjin Institute of Cardiology, Second Hospital of Tianjin Medical University, Tianjin 300211, China

**Keywords:** systemic sclerosis, arrhythmias, conduction disturbances

## Abstract

Systemic sclerosis (SSc) is an autoimmune disease characterized by skin and internal organ fibrosis and microvascular impairment, which can affect major organs, including the heart. Arrhythmias are responsible for approximately 6% of deaths in patients with SSc, and mainly occur due to myocardial fibrosis, which causes electrical inhomogeneity. The aim of this study was to determine the frequency of arrhythmias and conduction disturbances in SSc cohorts, and to identify the characteristics and risk factors associated with the occurrence of dysrhythmias in patients with SSc. A systematic literature review using PubMed, Embase, Web of Science and Scopus databases was performed. Full-text articles in English with arrhythmias as the main topic published until 21 April 2022 were included. Most prevalent arrhythmias were premature supraventricular and ventricular contractions, while the most frequent conduction disturbance was represented by right bundle branch block (RBBB). Elevated concentrations of N-terminal prohormones of brain natriuretic peptides (NT-pro BNP) were associated with numerous types of atrial and ventricular arrhythmias, and with the occurrence of RBBB. A lower value of the turbulence slope (TS) emerged as an independent predictor for ventricular arrhythmias. In conclusion, dysrhythmias are frequent in SSc cohorts. Paraclinical and laboratory parameters are useful instruments that could lead to early diagnosis in the course of the disease.

## 1. Introduction

Systemic sclerosis (SSc) is an autoimmune disease with heterogenous manifestations that can affect multiple organs, including the heart. Cardiac involvement, most often represented by heart failure, pericardial disease and rhythm disturbances, can be asymptomatic or occur with mild symptoms in the early stages of the disease. It is associated with a severe prognosis, and according to European Scleroderma Trials and Research group (EUSTAR) registries, 26% of the deaths in SSc patients are due to cardiac complications, mainly heart failure and arrhythmias [1]. Cardiac microvascular disease appears to be similar to the Raynaud phenomenon, with repeated episodes of ischemia and reperfusion of the myocardium, which lead to contraction band necrosis and fibrotic foci [2]. Myocardial fibrosis, which is supposed to follow the impairment of the microcirculation, is responsible for increasing the stiffness of the ventricular walls. This leads to systolic and diastolic dysfunction, causing the appearance of myocardial electrical inhomogeneities, an important mechanism of arrhythmogenesis. 

Cellular and molecular processes linked to the appearance of fibrosis are not yet fully understood, but several signaling molecules and extracellular factors, such as TGF beta (transforming growth factor-beta), reactive oxygen species and endothelin-1, are thought to be involved [3,4,5,6,7]. Endothelin-1 induces proliferation and differentiation of fibroblasts into myofibroblasts, and together with TGF-b, stimulates connective tissue growth factor (CTGF) secretion, which determines collagen production [4,5]. The TGF-b pathway is also associated with collagen overproduction through the increased expression of its receptors, such as TGF-b-RI and TGF-b-RII [6,7].

Arrhythmias and conduction disturbances are frequently encountered in SSc patients; according to an EUSTAR analysis, they are accountable for up to 6% of all-cause mortality in SSc patients [1]. Patchy myocardial fibrosis appears to also involve the conduction system, and it is responsible for bradyarrhythmia and conduction defects, whereas myocardial fibrosis is most likely the substrate for atrial and ventricular tachyarrhythmias [8,9,10]. Many authors have investigated the association of subclinical cardiac damage with paraclinical or laboratory elements; however, currently, arrhythmias are not routinely screened for, thus more data in this field are needed.

The aim of this review is to identify the characteristics and risk factors associated with the occurrence of arrhythmias and conduction disorders in patients with SSc, which could facilitate diagnosis and, ultimately, treatment earlier in the course of the disease, ideally in the subclinical stage.

## 2. Results

### 2.1. Data Extraction

**Figure 1:**
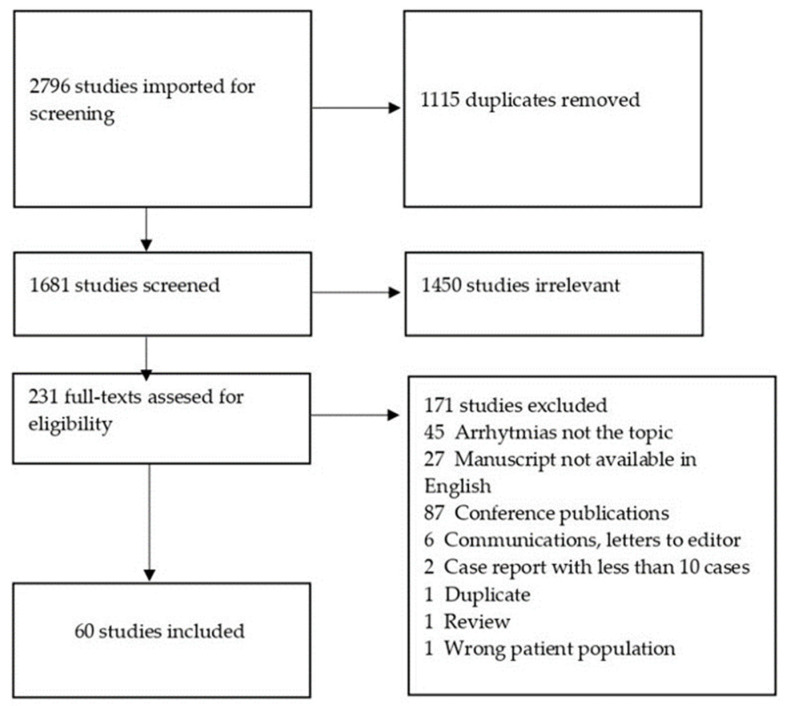
PRISMA flowchart.

A total of 2796 articles were identified, of which 1115 duplicates were removed (Figure 1). Following title and abstract screening, there were 231 articles for full-text screening included. If any conflicts emerged during the review process, they were resolved by discussion among the readers. Sixty articles were finally included in this systematic literature review [8,11,12,13,14,15,16,17,18,19,20,21,22,23,24,25,26,27,28,29,30,31,32,33,34,35,36,37,38,39,40,41,42,43,44,45,46,47,48,49,50,51,52,53,54,55,56,57,58,59,60,61,62,63,64,65,66,67,68,69].

Data from the 60 full-text articles included was extracted by two readers into a standardized data extraction form. Extracted data included demographic and disease features, presence and types of arrhythmias and conduction disturbances, as well as follow-up data regarding management and treatment of rhythm disorders were recorded. No randomized controlled trials (RCTs) were included. Quality assessment in prognostic studies and potential confounding measurements were determined using the guidelines proposed by Hayden et al. [70].

### 2.2. Characteristics of Studies

The demographic characteristics of the included patients are presented in Table 1. Six studies were retrospective, while most studies were prospective cohort studies, and had an overall good quality (Table 1). There were no randomized control trials among the included studies. Regarding the country the studies were carried out, we retrieved 35 from Europe, 12 from North America, 9 from Asia, 1 from Africa, 1 from Oceania and a multicentric study. Sample size ranged from 12 to 2778 patients with SSc. The mean age of patients ranged from 36.7 to 69.8 years, and most of the patients included were female, except for one study, in which the majority was represented by the male gender. Reported disease durations had mean values between 2 years and 12 years. Follow-up duration is reported either in months or in years, with ranges between 12.0 and 101.8 months and 1.0 and 10.5 years, respectively. Cutaneous involvement in SSc, either more severe diffuse skin thickening (dcSSc), with skin thickening proximal to the elbows and knees, or limited cutaneous thickening (lcSSc), confined to fingers and forearms, or without skin thickening, i.e., sine scleroderma, was also reported in the included studies. The disease subset of the included SSc patients showed predominant diffuse cutaneous SSc (dcSSc) in 18 out of 34 studies, and antibodies profile of SSc patients included 21 cohorts with anti-topoisomerase I antibodies (ATA) predominance, nine with anti-centromere antibodies (ACA) and two with anti-RNA polymerase III antibodies (ARA).

### 2.3. Characteristics of Studied Cohorts—General Cardiovascular Risk Factors 

The risk of developing arrhythmic complications in SSc patients is increased by the presence of general cardiovascular risk factors, such as arterial hypertension, dyslipidemia, diabetes, obesity, smoking status and coronary artery disease (CAD). The highest prevalence in the studied cohorts for arterial hypertension was 50% (9/18) [35], 19.5% (8/41) for diabetes [38], 41.5% (17/40) for dyslipidemia [48], 21% (15/78) for obesity [54] and 14% (11/78) for CAD [54]. Smoking status was mentioned in few studies and, moreover, not all authors reported the number of active or former smokers. The highest prevalence was 50.3% (143/284), with 118 former and 25 active smokers [44] (Table 2).

### 2.4. Arrhythmias—Description and Risk Factors

#### 2.4.1. Atrial Arrhythmias

Supraventricular premature beats (SPB) were one of the most frequent manifestations highlighted on the surface EKG or Holter EKG. Its prevalence varied in the studied populations from 2.6% (7/265) in a study conducted by Draeger [24] to 90% (28/31), with a mean number of 1430 (±6185) SPB, in a study that assessed myocardial fibrosis by cardiac magnetic resonance imaging in SSc patients with no history of cardiovascular disease [57]. However, it should be specified that there was heterogeneity in reporting the SPB frequency depending on the study. Some authors presented the number of patients with isolated SPB, regardless of their number, in 24 h [18,29], while other authors only reported SPB if there were more than 100/24 h or 1000/24 h [21,48,49,57]. 

Prevalence of supraventricular tachycardia (SVT) ranged from 0.9% (1/110) to 51% (23/45) [13,49], atrial flutter (AFL) from 2.1% (1/46) to 40% (10/25) [20,32] and atrial fibrillation (AFib) from 1.8% (1/53) to 36.7% (18/49) in the selected cohorts [29,33]. Some authors reported the cumulative frequency of AFL and AFib. Moreover, Mercurio et. al. noticed, in their study, the presence of 10/201 patients with flutter fibrillation [44] (Table 3). The same study showed that the occurrence of atrial arrhythmias was associated with higher levels of N-terminal prohormones of brain natriuretic peptides (NT-proBNP), as well as higher pressures in both atria, as suggested by cardiac catheterization [44]. Elevated levels of NT-proBNP and high sensitivity troponin I (hs-TnI) were significantly associated with arrhythmias in a study by Bissel et al., in which two out of 19 patients had episodes of SVT, two had AFL, three had AFib and two had nonsustained ventricular tachycardia (NSVT) [11]. 

Moreover, B-type natriuretic peptide (BNP) with a cutoff level of 104.5 pg/mL appeared to be the only significant predictor for AFib in a prospective study conducted by Giallafos, in which 18/49 SSc patients with a mean follow-up of 72 ± 24 months developed AFib [34].

#### 2.4.2. Ventricular Arrhythmias

Prevalence of premature ventricular contractions (PVC) ranged from 3.7% (1/27) to 100% (39/39) in a SSc cohort with prolonged QT, for which the number of PVC on the Holter EKG was significantly higher (*p* = 0.04) in patients with dcSSc compared to lcSSc [15,59]. Of the studies that reported mean PVC, this value varied between 197 ± 527 and 2046.1 ± 6027.8 [21,57]. As in the case of SPB, the reporting of PVC varied depending on the study, but most authors classified ventricular arrhythmias according to the Lown class. PVC occurring in repeating patterns were also frequently observed in the studied cohorts. The number of cases with bigeminy and trigeminy had the highest percentages in a study conducted by Muresan, 26.6% (8/30), which detected myocardial fibrosis by delayed-enhancement magnetic resonance imaging (DE-MRI) in 83.3% subjects, out of which, 18 had ventricular arrhythmias or conduction disorders [47]. The highest frequency of couplets and triplets, 39% (7/18) emerged from a prospective study that included SSc patients who underwent myocardial biopsy for cardiac fibrosis [46] (Table 4). Cardiovascular events represented by pacemaker implantation, implantable cardioverter defibrillator, ablation or sudden cardiac death in the cohorts included in this review are presented in Table 4.

Most cases of NSVT (15/53) were encountered in a study that assessed the relationship between symptoms and noninvasive evaluation of patients with arrhythmias [29]. Sustained ventricular tachycardia was less common in the studied cohorts.

Two studies showed an association between skin involvement, evaluated by modified Rodnan skin score (mRSS). The number of PVC directly correlated with mRSS in one of the studies, while the other showed an association of supraventricular arrhythmias with higher skin scores [21,49].

NT-proBNP and high-sensitivity cardiac troponin (hs-Tn) were the laboratory parameters that correlated with the presence of ventricular arrhythmias in multiple studies. One of them demonstrated that the number of SPB and PVC correlated with hs-cTnT and NT-proBNP levels [21], and another showed that a concentration of NT-proBNP in serum >287 pg/mL has a sensitivity of 55% and a specificity of 93% in predicting the occurrence of complex ventricular arrhythmias on a Holter monitor [49].

One study showed that the number of SPBs and PVC inversely correlated with the left ventricular ejection fraction (LVEF) in echocardiography [21].

HRV (heart rate variability) and HRT (heart rate turbulence) were recently proposed as instruments that could be used for risk stratification, especially in patients with ventricular arrhythmias [13]. One study showed that HRT parameters, more precisely, the median value of TS (turbulence slope), was significantly lower in patients with Lown class IV A (couplets) and Lown class IV B (NSVT), than in patients without ventricular arrhythmias Lown class IV A and IV B. Lower TS values emerged as an independent predictor of ventricular arrhythmia Lown IV occurrence [13]. However, these authors did not find any significant differences in the values of TO (turbulence onset). In a study by Othman et. al, total skin score showed significant correlation with all arrhythmic parameters of HRV [52].

### 2.5. Conduction Disturbances—Description and Risk Factors

The conduction disturbance with the highest prevalence in the studied cohorts was the right bundle branch block (RBBB). Most cases were reported in a study published in 2018, whose aim was to determine whether there was any correlation between conduction disorders, ventricular arrhythmias and myocardial fibrosis in SSc patients, with a percentage of 6.6% for complete and 13.3% for incomplete RBBB, a total of 6/30 subjects [47]. Not all authors reported whether the patients had complete or incomplete RBBB. Other relatively frequent conduction disorders were left bundle branch block (LBBB), left anterior fascicular block (LAFB) and atrioventricular block (AVB). Among SSc patients with atrioventricular block, the most frequent was first-degree AVB, ranging from 1.3% (1/72) [45] to 55% (22/40) [41], while third-degree AVB was the least common, with the highest percentage, reported in a study by Bienias, of 2.7% (2/74) [17] (Table 5).

One study showed that elevated concentrations of hs-cTnT (high-sensitivity cardiac troponin T) was associated with a higher occurrence of RBBB on the ECG [12]. In a study by Follansbee et al., RBBB and isolated LAFB were associated with normal left ventricular function, whereas LBBB and bifascicular block (RBBB with LAFB) were associated with abnormal left ventricular function [8]. LPFB (left posterior fascicular block) was only reported in one study [58] (Table 5).

### 2.6. Treatment and Outcome

Regarding treatment, most frequently, the patients in the studies were treated with calcium channel blockers (CCBs) or beta blockers. Calcium channel blockers in SSc are considered to have a vasodilator effect that improves both myocardial perfusion with a decrease in the risk of cardiovascular complications, as well as peripheral circulation, with the improvement of Raynaud’s phenomenon (RP) [71]. Contrarywise, the negative effect of beta blockers in Raynaud’s phenomenon is well known. However, the use of metoprolol together with CCBs was reported to reduce the symptoms in patients with RP. Amiodarone was most frequently reported in studies, followed by propafenone and sotalol, each mentioned in one study.

Very few studies evaluated the use of immunosuppressive treatment for cardiac fibrosis, but the most frequently used were cyclophosphamide and mycophenolate mofetil. Most cases were reported in a study published in 2018 by Hu et al., including 250/448 patients with cyclophosphamide and 28/448 with mycophenolate mofetil [37].

Few studies reported cardiovascular events such as arrhythmias that required implantable cardioverter defibrillator (ICD), conduction disturbances that required cardiac pacing or cases of sudden cardiac death (SCD). ICD is considered in patients that may develop malignant ventricular arrhythmias, in order to prevent SCD. Most cases (10/150) are described in The Scleroderma Arrhythmia Clinical Utility Study (SAnCtUS) cohort, a prospective multicentric study, which aimed to determine the most useful predictors for ventricular arrhythmias in SSc patients by cardiac magnetic resonance [43]. Pacemaker implantation is indicated in the treatment of complete heart blocks and other bradyarrhythmias, and it had the highest prevalence in the reviewed studies, reaching 5.1% (4/78) [54]. Catheter ablation was reported in one study in 2/78 patients with tachyarrhythmias [54].

Most cases of SCD were observed in the study that also presented the highest frequency of ventricular tachycardia, with 12/25 cases [32].

## 3. Methods

### 3.1. Research Question and Search Strategy

A systematic literature review was conducted using the electronic PubMed, Embase, Web of Science and Scopus databases in accordance with the Preferred Reporting Items for Systematic Reviews and Meta-Analyses (PRISMA) statement. The search included studies published until 21 April 2022 with no limit for the starting date. The research question was constructed according to the PICO (Population, Intervention, Comparison, Outcome) method. Population was represented by SSc patients; intervention by diagnostic tests, including ECG, Holter ECG, echocardiography and laboratory tests associated with the occurrence of rhythm disorders; and outcome was defined as arrhythmias and conduction disturbances.

A combination of MeSH terms (Medical Subject Headings) and SSc- and arrhythmia-related keywords, such as (but not limited to) “systemic sclerosis” and “scleroderma” and “arrhythmias”, “atrial flutter”, “atrial fibrillation”, “premature ventricular contraction”, “ventricular tachycardia”, respectively, and the categories “screening”, “treatment” and “diagnosis” were used.

### 3.2. Selection of Studies 

For reference screening, the platform Covidence, a tool dedicated for screening and data extraction, was used. The selection of studies was carried out by four independent reviewers, who screened the references in pairs of two. Randomized clinical trials, prospective and retrospective observational studies, as well as case series comprising more than 10 SSc patients written in English with arrhythmias as the main topic, were included. Articles that did not have systemic sclerosis and arrhythmias as a topic, studies evaluating pediatric patients, and basic/genetic and nonclinical studies, were excluded. Literature reviews were excluded from the selection, but they were used for additional references. 

## 4. Discussion

Arrhythmias and conduction disturbances are common in SSc patients. They are associated with an increased risk of mortality, which makes their detection before the onset of symptoms very important. Most prevalent changes on the Holter monitor or surface EKG are premature supraventricular and ventricular contractions. However, it is difficult to specify the frequency with which they appear in the SSc population, considering the heterogeneous manner in which they were reported, some authors considering SPBs or PVC relevant only over a certain frequency in 24 hours. Supraventricular tachyarrhythmias comprise a wider group of arrhythmias. Few authors specified which type of supraventricular arrhythmia was encountered in the studied cohorts, most reporting the total number of subjects with this type of dysrhythmia. However, it had a high prevalence, followed by AFib and AFL, which had a relatively similar frequency of occurrence.

Literature data suggest that extension of skin involvement, determined by mRSS, is associated with the occurrence of arrhythmias, specifically supraventricular arrhythmias and PVCs. Laboratory parameters such as NT-proBNP, hs-TnI and hs-TnT significantly correlated with the presence of dysrhythmia. Elevated levels of NT-proBNP and hs-TnI showed an association with both supraventricular and ventricular arrhythmias, while hs-TnT correlated with the number of PVC and SPBs. Moreover, Muresan et al. showed that a concentration of NT-proBNP in serum >287 pg/mL has a sensitivity of 55% and a specificity of 93% in predicting the occurrence of complex ventricular arrhythmias [49]. 

Regarding the echocardiographic variables, one study showed that number of SPBs and PVC were inversely correlated with LVEF [21]. HRT and HRV parameters of the Holter monitor were recently proposed as useful tools for risk stratification in patients prone to ventricular arrhythmias. From the studies included in our review, only a lower value of TS, an HRT parameter, emerged as an independent predictor for ventricular arrhythmias, with a median significantly lower in patients with Lown class IV A (couplets) and Lown class IV B (nonsustained ventricular tachycardia), compared to patients without [13].

Conduction disorders were most frequently represented by right bundle branch block. We did not exclude from the search patients with pulmonary arterial hypertension, which could influence the higher frequency of RBBB in the studies we evaluated. Third-degree AVB and LAFB were the least frequent in the studied cohorts. Higher levels of hs-cTnT were associated with the presence of RBBB on the surface ECG [12].

### Study Limitations

Limitations of our systematic literature review are represented by the exclusion of studies of which the full text was not available in English, which could potentially lead to the loss of significant data for this review and limited our access to the full texts of a few older articles. Moreover, the heterogenous reporting of data for SPB and PVCs, as well as the lack of reporting of data for supraventricular tachycardia, could lead to the underdetection of these common arrhythmias. Due to the fact that SSc is a rare condition, most of the included studies had a limited sample size of patients. Another limitation of our review is represented by the fact that the etiology of arrhythmias in SSc patients cannot be accurately determined. For the majority of the evaluated studies, SSc patients with pre-existing heart conditions, antiarrhythmic therapy or other conditions that could lead to the occurrence of arrhythmias and conduction disorders (e.g., dyselectrolytemia, renal involvement), were excluded. Moreover, it is worth mentioning that we did not exclude the studies of patients with PAH, this also being an important cause of arrhythmias in SSc. Diabetes mellitus, arterial hypertension and coronary artery disease, conditions that could influence the occurrence of dysrhythmia, were accounted for in several studies.

Data from literature related to arrhythmias and conduction disorders in SSc patients are insufficient. Although there were several studies included in the present review that evaluated cardiac damage in SSc, few of them reported the frequency of arrhythmias within these cohorts, or other clinical and laboratory elements that could contribute to early diagnosis in the case of these patients. Identification of cardiac damage in subclinical stages in SSc patients through laboratory, EKG, Holter monitor and echocardiographic parameters that are inexpensive and readily available represents a new target in SSc evaluation; however, more studies are needed. Management of arrhythmias and conduction disturbances, including immunosuppressive treatment for cardiac fibrosis, also warrants further exploration.

## 5. Conclusions

In conclusion, arrhythmias and conduction disorders have an increased prevalence in SSc patients. Laboratory elements such as NT-proBNP, hs-TnI and hs-TnT correlate with the presence of both supraventricular and ventricular arrhythmias, and increased values of hs-TnT were associated with a higher frequency of RBBB occurrence. The number of SPBs and PVC inversely correlated with LVEF in echocardiography, and LBBB and bifascicular block were associated with abnormal left ventricular function. Among the Holter parameters evaluated in the included studies, only the low TS value proved to be an independent predictor for ventricular arrhythmias. Currently, there are few sources of literature data on arrhythmic risk stratification in SSc patients. Extensive studies evaluating these new parameters, as well as other paraclinical and laboratory elements, are necessary for the determination of subclinical damage, early diagnosis and a faster therapeutic intervention during the course of the disease.

## Figures and Tables

**Table 1:** Characteristic of cohorts.

Author (Reference)	Country	Overall Quality	No. SSc Patients	Mean Age ± SD	Disease Duration (Years) ± SD	Predominant Gender (%)	Predominant Subset (%)	Predominant Antibodies Profile (%)	mRSS Mean/Median ± SD
Alba [55]	Spain	6	1037	51.0 ± 15		88	lcSSc (60.2)	ACA (43.6)	-
Anvari [66]	Austria	5	18	56.3 ± 11.2	9.7 ± 6.3	88.8			-
Arakkal [67]	India	5	28	36.7	2.9	96.4	dcSSc (60.7)	ATA (50)	-
Assassi [68]	USA	6	250	48.85 ± 13.7	2.6 ± 1.64	84	dcSSc (57.4)	ARA (22.9)	-
Baek [69]	Republic of Korea	5	303	49.6 ± 14.7	-	86.4	-	-	-
Biełous-Wilk [15]	Poland	4	27	55.2 ± 11.3	8.4 ± 5.3	66.6	lcSSc (51.85)	-	-
Bienias [16]	Poland	6	45	54.6 ± 14.7	11.7 ± 11.4	88.8	lcSSc (51)	-	-
Bienias [17]	Poland	6	74	51.9 ± 13.1	6.0	90.5	lcSSc (56.8)	ATA (54)	-
Bissel [11]	UK	6	19	53.0	7.5	63	-	ACA (32)	-
Bosselo [12]	Italy	6	195	56.0	7.0	88.2	-	ATA (49.2)	6.0
Butrous [18]	UK	4	28	48 ± 13	11 ± 6	75	-	-	-
Butt [19]	Denmark	5	2778	55 ± 15	-	76	-	-	-
Clements [20]	USA	5	46	50.0	9,0	86.9	-	-	-
De Luca [21]	Italy	6	100	56.1 ± 15.2	10.2 ± 9.3	85	dcSSc (55)	ATA (46)	-
De Luca [22]	Italy	6	19	54.0	-	78.9	dcSSc (52.6)	ATA (52.6)	8.0—median
De Luca [23]	Italy	6	12						
Draeger [24]	USA	5	265	48.7 ± 13.2	2.5 ± 1.6	84	dcSSc (56.6)	ARA (19.6)	-
Dumitru [25]	UK	6	74	57.0	8.0	85	lcSSc (68)	ACA (35)	2.0
Edigin [26]	USA	4	750	69.8	-	80	-	-	-
Escudero [27]	USA	3	60	-	-	76.6	-	-	-
Fernández-Codina [28]	Spain	6	393	50.8 ± 15.4		90	lcSSc (59)	ACA (40)	-
Ferri [29]	Italy	5	53	50.0	7.7	75.4	dcSSc (64)	-	-
Ferri [30]	Italy	5	35	48.6 ± 11	8.3 ± 6.0	91.4	lcSSc (88.5)	ACA (40)	-
Ferri [31]	Italy	6	30	45.2 ± 9	10 ± 9.9	86.6	lcSSc (43)	ATA (63)	-
Follansbee [8]	USA	5	102	51 ± 13	7.6 ± 8.1	80.3	dcSSc (52)	-	-
Follansbee [32]	USA	5	39	45.0		76	dcSSc (46.1)	-	-
Gialafos [33]	Greece	6	69	50.8 ± 12.5	8.7 ± 6.3	91.3	dcSSc (60.8)	ATA(57.9)	-
Giallafos [34]	Greece	6	49	50.15 ± 9.25		87.8	-	-	-
Gurtner [35]	Germany	4	18	46.5	5.5	83.3	-	-	-
Henein [36]	UK	6	34	49 ± 12	-	73.5	lcSSc (44.1)	-	-
Hu [37]	China	6	448	-	7.0	90.4	lcSSc (56.7)	ATA (46.8)	6.0
Javinani [38]	Iran	5	41	47.48 ± 11.57	7.0	82.9	lcSSc (56.1)		2.0—median
Kaburaki [39]	Japan	4	86	47.5	-	87.2	-	-	-
Kostis [40]	USA	6	183	49.0 ± 13.0		79	dcSSc (56.8)	-	-
Kramarz [41]	Poland	4	40	54.8 ± 12.9	8.9 ± 8.3	100	-	ACA (55)	5.6 ± 5.8
Lui [42]	USA	4	169	-	-	-	-	-	-
Mavrogeni [43]	Multicentric	6	150	54.3 ± 13.8	9.0	84	dcSSc (59.3)	ATA (60.4)	4.0
Mercurio [44]	USA	6	201	-	-	-	-	-	-
Morelli [45]	Italy	5	72	48.0	10.0	87.5	dcSSc (59.7)	-	-
Mueller [46]	Germany	6	25	46.0 ± 11.0	2.0	32	dcSSc (72)	ATA (52)	16.0—median
Muresan [47]	Romania	6	30	48.6 ± 11.1	-	89.6	dcSSc (53.3)	ATA (46.6)	12.2
Muresan [48]	Romania	6	40	50.05 ± 12.12	-	92.5	-	-	9.0
Muresan [49]	Romania	6	110	52.57 ± 12.34	-	91.8	dcSSc (57.3)	ATA (43.6)	10.56
Niklas [50]	Poland	4	69	56.6 ± 13.1	-	88.4	dcSSc (84.0)	-	-
Nordin [51]	Sweden	6	110	61.7 ± 12.4	9.4	81	lcSSc (78)	ACA (30)	6.0
Nussinovitch [14]	Israel	6	21	45.9 ± 12.0	-	90.4	-	-	-
Othman [52]	Egypt	6	30	36.8 ± 6.5	4.4 ± 1.8	83	dcSSc (53)	ATA (50)	14.1 ± 7.2
Poormoghim [53]	Iran	5	58	40.8 ± 13.7	9.97	53	lcSSc (59.6)	-	16.6
Radwan [54]	USA	5	78	56.1 ± 15.7		91	lcSSc (83)	ACA (83)	-
Roberts [56]	USA	2	50	49.0	9.3	44	-	-	-
Ross [57]	Australia	6	34	55.1 ± 7.54	9.71 ± 7.33	74	dcSSc (58)	ATA (39)	10—median
Sano [58]	Japan	5	40	58.35 ± 14	106 ± 113	87.5	lcSSc (63.5)	ACA (12)	-
Saramet [59]	Romania	4	39	56.41 ± 11.26		74.3	dcSSc (79.5)	ATA (43.5)	-
Sergiacomi [60]	Italy	6	20	54.7 ± 13.7	12.0 ± 10.7	100	lcSSc (60)	ACA (50)	-
Tzelepis [61]	Greece	4	41	-	-	78.0	-	ATA (78.0)	-
Valentini [62]	Italy	4	601	56 ± 13	10 ± 9	88.0	lcSSc (78.5)	ACA (37.7)	-
Wangkaew [63]	Thailand	4	114	51.4 ± 8.5	11.7 ± 8.8	60.5	dcSSc (78.9)	ATA (78)	
Wranicz [64]	Poland	4	22	52	5	77.2		ATA (59.0)	
Yiu [65]	Netherlands	5	104	54 ± 12	8.6 ± 6.3	76.9	dcSSc (50.9)	ATA (35)	

Overall quality: 6–5, high; 4–5, moderate; 2–3, low; no, number; dcSSc, diffuse cutaneous systemic sclerosis; lcSSc, limited cutaneous systemic sclerosis; ACA, anti-centromere antibodies; ATA, anti-topoisomerase I antibodies; ARA, anti-RNA polymerase III antibodies; mRSS, modified Rodnan skin score.

**Table 2:** Characteristics of SSc patients from cohorts—general cardiovascular risk factors.

Author (Ref.)	Smoking Status, n/N (%)	Dyslipidemia, n/N (%)	Diabetes, n/N (%)	Arterial Hypertension, n/N (%)	Obesity, n/N (%)	CAD, n/N (%)
Baek [69]	-	35/303 (11.5)	24/303 (7.9)	90/303 (29.7)	2/303 (0.6)	8/303 (2.6)
Bissel [11]	11/58 (18.9), former	-	-	0/19	0/19	-
Bosselo [12]	27/195 (13.8)	40/195 (20.5)	11/195 (5.6)	26/195 (13.3)	14/195 (7.1)	-
Butt [19]	-	-	-	1320/2778 (47.5)	-	-
Draeger [24]	45/265 (16.9)	-	-	67/265 (25.2)	-	9/265 (3.3)
Dumitru [25]	6/74 (8.1)	3/74 (4.0)	-	8/74 (10.8)	-	-
Giallafos [34]	5/49 (10.2)	-	1/49 (2.0)	6/49 (12.2)	-	-
Gurtner [35]	3/18 (16.6)	-	-	9/18 (50)	-	-
Hu [37]	-	-	-	47/448 (10.4)	-	9/448 (2.0)
Javinani [38]	4/41 (9.7)	-	8/41 (19.5)	9/41 (21.9)	-	-
Mercurio [44]	143/284 (50.3) 118 (41.5), former 25 (8.8), active	94/303(31.0)	21/317 (6.6)	95/317 (30.0)	-	19/317 (5.9)
Mueller [46]	-	5 /25 (20.0)	1/25 (4.0)	5/25 (20.0)	-	-
Muresan [47]	-	9/30 (30.0)	-	2/30 (6.6)	-	-
Muresan [48]	-	17/40 (42.5)	-	7/40 (17.5)	-	-
Muresan [49]	-	42/110 (38.1)	4/110 (3.6)	30/110 (27.2)	-	9/110 (8.1)
Radwan [54]	36/78 (46.1) 12 (15.3), current 24 (30.7), former	27/78 (34.6)	2/78 (2.5)	30/78 (38.4)	15/78 (19.2)	11/78 (14.1)
Ross [57]	2/31 (6.4), current	-	1/31 (3.2)	2/31 (6.4)	-	-
Sano [58]	-	-	-	11/40 (27.5)	-	-
Sergiacomi [60]	2/18 (11.1)	8/18 (44.4), hypercholesterolemia 6/18 (33.3), hypertriglyceridemia	1/18 (5.5)	-	-	-
Valentini [62]	127/601 (21.1)	-	-	139/601 (23.1)	-	-
Wranicz [64]	-	-	-	2/22 (9.0)	-	-

CAD, coronary artery disease; N, total number of patients; n, number of patients with cardiovascular risk factors; %, percentage of patients with cardiovascular risk factors.

**Table 3:** Frequency of atrial arrhythmias in SSc cohorts.

Author (Ref.)	PAH Excluded	Study Designed for PAH	Patients with SPBs, n/N (%)	Mean SPBs	Patients with SVT, n/N (%)	Patients with Atrial Flutter, n/N (%)	Patients with Atrial Fibrillation, n/N (%)
Anvari [66]	No	-	2/18 (11.1) > 720/day	-	6/18 (33.3)	-	1/18 (5.5)
Baek [69]	-	-	-	-	-	-	7/303 (2.3)
Bielous-Wilk [15]	No	-	-	-	4/27 (14.8)	-	1/27 (3.7)
Bienias [16]	-	-		-	23/45 (51.1), nonsustained SVT 2/45 (4.4), sustained SVT	2/45 (4.4)-AFib AFL	-
Bienias [17]	-	-	19/74 (25.6)	-	37/74 (50), nonsustained SVT		4/74 (5.4)
Bissel [11]	Yes	-		-	2/19 (10.5)	2/19 (10.5)	3/19 (15.7)
Butrous [18]	-	-	4/28 (14.2)	-	-	-	2/28 (11.0)
Butt [19]	No	-	-	-	-	-	235/2778 (8.4)-FU, 77 (2.7), baseline AFib + AFL
Clements [20]	No	-	4/46 (8.6)		13/46 (28.2)	1/46 (2.1)	1/46 (2.1)
De Luca [21]	No	-	49/100 (49.0)	798.9 (1835.6)	14/100 (14.0)	-	4/100 (4.0)
Draeger [24]	No	-	7/265 (2.6)	-	-	-	-
Dumitru [25]	Yes	-	-	-	1/74 (1.3)	2/74 (2.7)	3/74 (4.0)
Escudero [27]	-	-	-	-	1/60 (1.6)	-	-
Ferri [29]	No	-	26/53 (49.0)	-	8/53 (15.0)	-	1/53 (1.8)
Follansbee [32]	-	-	-	-	5/25 (20)	10/25 (40)	5/25 (20)
Gialafos [34]	-	-	-	-	-	-	18/49 (36.7)
Kostis [40]	No	-	111/183 (60.6)	-	38/183 (20.7)	-	-
Mavrogeni [43]	-	-	-	-	9/150 (6.0)	-	12/150 (8.0)
Mercurio [44]	-	Yes		-	4/201 (1.9), atrial ectopic tachycardia	9/201 (4.4)	19/201 (9.4) 10/201 (4.9), flutter fibrillation
Muresan [47]	-	-	10/30 (33.3) > 100 isolated SPBs	-	-	-	-
Muresan [48]	-	-	10/40 (25) > 100 SPBs	-	-	-	-
Muresan [49]	No	-	79/110 (71.8) 56/110 (50.9), coupled, SPBs, 73/110 (66.3), triplets/runs of	-	1/110(0.9)	-	AFib/AFL/tachycardia, 2/110 (1.8)
Nordin [51]	-	-	-	-	-	-	AFib, 1/19 (5.2)
Othman [52]	-	-	-	-	2/30(6.6)	-	
Radwan [54]	No	-	-	-	-	-	10/78 (12.8), AFib or AFL baseline 13/78 (16.6), FU
Roberts [56]	-	-	-	-	17/50 (34%)	-	1/50 (2.0), AFib, 1/50 (2.0), AFL
Ross [57]	Yes	-	28/31 (90.3)	1430 (6185)	9/31 (29.0)	-	1/31 (3.2)
Tzelepis [61]	No	-	3/36 (8.3)	-	2/36 (5.5)	-	2/36 (5.5), AFib
Wranciz [64]	-	-	-	-	2/22 (9.0)	-	-

SPB, supraventricular premature beats; AFib, atrial fibrillation; FU, follow-up; AFL, atrial flutter; SVT, supraventricular tachycardia; N, total number of patients; n, number of patients with atrial arrhythmias; %, percentage of patients with atrial arrhythmias.

**Table 4:** Frequency of ventricular arrhythmias and cardiovascular events in SSc cohorts.

Author	PAH Excluded	Study Designed for PAH	Patients with PVC, n/N (%)	Mean PVC ± SD	Patients with Bigeminy/Trigeminy, N (%)	Patients with Couplets, n/N (%)	Patients with VT, n/N (%)	Events, n/N (%)
Anvari [66]	No	-	6/18 (33.3)	-	--	-	5/18 (27.7), NSVT	-
Bielous-Wilk [15]	No	-	1/27 (3.7)	-	Bigeminy 2/27 (7.4)	2/27 (7.4)	-	-
Bienias [16]	-	-	-	-	-	13/45 (28.8)	7/45 (15.5)	-
Bienias [17]	-	-	18/74 (24.3)	-	Bigeminy/trigeminy/couplets, 26/74 (35.1)	-	10/74 (13.5)	-
Bissel [11]	Yes	-	-	-	-	-	2/19 (10.5), NSVT	1/19 (5.2), pacemaker
Butrous [18]	-	-	Present, not determined	-	-	1/28 (3.5)	1/28 (3.5)	pacemaker/ICD 18/1778 (1.0), baseline 48/2778 (2.6) (FU)
Clements [20]	No	-	4/46 (8.6)	-	-	4/46(8.6)	6/46 (13.0)-NSVT 1/46 (2.1), VT	-
De Luca [21]	No	-	42/100 (42.0)	2046.1 (6027.8)	Bigeminy, 9/100 (9.0)	-	11/100 (11.0)	2/100 (2.0)-ICD 5/100 (5.0)-SCD
De Luca [22]	-	-	5/19 (26.3)	-	Bigeminy, 3/19 (15.7)	-	3/19 (15.7), VT	-
Draeger [24]	No	-	8/265 (8.0)	-	-	-	-	-
Dumitru [25]	Yes	-	-	-	-	-	4/74 (5.4), NSVT	-
Escudero [27]	-	-	2/60 (3.3)	-	-	-	-	-
Ferri [29]	No	-	-	-	-	-	15/53 (28.3)	1 (1.8), SCD
Ferri [30]	-	-	4/35 (11.4)	-	-	-	-	
Follansbee [32]	-	-	12/25 (48)	-	-	-	2/25 (8), NSVT 5/25 (20), VT	12 (48), SCD
Gialafos [33]	-	-	-	-	-	6/69 (8.6)	5/69 (7.2), NSVT	-
Gurtner [35]	-	-	Present, not determined	-	-	-	1/18 (5.5), NSVT	
Kostis [40]	No	-	46/183 (25.1)	-	-	-	12/183 (6.5)	1 (0.5), pacemaker SCD, 12/183 (6.5)
Mavrogeni [43]	No	-	-	-	Bigeminy/trigeminy/quadrigeminy, 25/150 (16.6)	45/150 (30)	7/150 (4.6), baseline 10/150 (6.6)—endpoint	10/15 (6.6), ICD
Morelli [45]	No	-	3/72 (4.1)	-	-	-	-	-
Mueller [46]	No	-		-	-	7/18 (38.8) couplets and triplets	5/18 (27.7), NSVT or ventricular salvos	3/18 (16.6), ICD shocks FU 6/18 (33.3), all-cause deaths 4/18 (22.2), CV deaths 3/18 (16.6), SCD
Muresan [47]	-	-	3/30 (10.0) isolated PVC	-	8/30 (26.6), bigeminy/trigeminy	3/30 (10.0), couplets	2/30 (6.6), NSVT	-
Muresan [48]	-	-	10/40 (25.0) > 100 PVC	-		7/40 (17.5)	1/40 (2.5), NSVT	-
Muresan [49]	No	-	76/110 (69.0), isolated PVC	-	Polymorphic/bigeminy/trigeminy, 18/110 (16.3)	18/110 (16.3)	7/110 (6.3), NSVT	-
Nussinovitch [14]		-		-	-	-	1/21 (4.7), NSVT FU	-
Othman [52]		-	10/30 (33.3) > 10 PVCs/h	798.75 (2654.2)	-	-	-	-
Radwan [54]	No	-	5/78 (6.4), baseline 16/78 (20.5), FU	-	-	-	-	2/78 (2.5), ablation FU 4/78 (5.1), pacemaker FU 4/78 (5.1), ICD
Roberts [56]	-	-	Present, not determined	-	1/50 (2.0), bigeminy	6/50 (12.0)	5/50 (10.0)	-
Ross [57]	Yes	-	17/31 (54.8)	197 (527)	6/31 (19.3), bigeminy 3/31 (9.6), trigeminy	2/31 (6.4), couplets 1/31 (3.2), triplets	1/31 (3.2), NSVT	-
Saramet [59]	-	-	39/39 (100.0)	-	-	3/39 (7.6), couplets	3/39 (7.6), NSVT	-
Tzelepis [61]	No	-	12/36 (33.3)	-	-	-	2/36 (5.5), NSVT	-
Wranicz [64]	-	-	-	-	-	2/22(9.0), couplet	3/22 (13.0), NSVT	-
Yiu [65]	No	-	19/100 (19.0)	-	-	-	9/100 (9.0), NSVT	-

PAH, pulmonary arterial hypertension; PVC, premature ventricular contractions; FU, follow-up; VT, ventricular tachycardia; NSVT, nonsustained ventricular tachycardia; SCD, sudden cardiac death; ICD, implantable cardioverter defibrillator; N, total number of patients; n, number of patients with ventricular arrhythmias; %, percentage of patients with ventricular arrhythmias.

**Table 5:** Frequency of conduction disturbances in SSc cohorts.

Author	PAH Excluded	Study Designed for PAH	RBBB n/N(%)	LBBB n/N (%)	AVB n/N (%)	LAFB n/N (%)	LPFB n/N (%)
Anvari [66]	No	-	2/18 (11.1)	2/18 (11.1)	1/18 (5.5)—AVB I	-	-
Bielous-Wilk [15]	No	-	1/27 (3.7) incomplete RBBB	-	-	6/27 (22.2)	-
Bienias [16]	-	-	-	-	-	6	-
Bienias [17]	-	-	Present, not determined	-	4/74 (5.4)—AVB I 7/74 (9.4)—AVB II 2/74 (2.7)—AVB III		-
Bissel [11]	-	-	1/19 (5.2)	1/19 (5.2)	-	1/19 (5.2)	-
Bosselo [12]	-	-	11/195 (5.6)	-	-	-	-
Butrous [18]	-	-	5/28 (17.8)	-	2/28 (7.1)—AVB II	-	-
Clements [20]	No	-	-	-	3/46 (6.5)—AVB I	8/46 (17.3)	-
De Luca [21]	No	-	19/100 (19.0)	4/100 (4.0)	4/100 (4.1)—AVB I		-
Draeger [24]	No	-	7/265 (2.6)	11/265 (4.1) 2 (0.7), complete, 9 (3.3), incomplete	14/265 (5.2)—AVB I	3/265 (1.1)	-
Escudero [27]	-	-	7/60 (11.6)	4/60 (6.6)	1/60 (1.6)—AVB III	-	-
Fernández-Codina [28]	-	-	25/393 (6.3)	-	-	-	-
Ferri [29]	-No	-	4/53 (7.5)	-	2/53 (3.7)—AVB I 7/53 (13.2)—AVB II	3/53 (5.6)	-
Follansbee [8]	-	-	3/102 (2.9)	3/102 (2.9)	-	10/102 (9.8)	-
Follansbee [32]	-	-	4/25 (16.0)	2/25 (8.0)	-	13/25 (52.0)	-
Gialafos [33]	-	-	9/69 (13.0)	4/69 (5.7)	-	4/69 (5.7)	-
Gurtner [35]	-	-	-	-	-	1/18 (5.5)	-
Henein [36]	-	-	1/34 (2.9)	-	-		-
Javinani [38]	-	-	4/41 (9.7)	-	-	5/41 (12.1)	-
Kostis [40]	No	-	-	-	1/183 (0.5)—AVB III		-
Kramaraz [41]		-	-	-	22/40 (55.0)—AVB I 15/40 (37.5)—AVB II		-
Morelli [45]	No	-	13/72 (18.0), 3/72 (4.1), complete; 10 (13.8), incomplete	-	1/72 (1.3)—AVB I	10/72 (13.8)	-
Muresan [47]	-	-	6/30 (20.0), 2/30 (6.6) complete, 4/30 (13.3), incomplete	1/30 (3.3), incomplete	-	4/30 (13.3)	-
Muresan [49]	No	-	13/110 (11.8); 4/110 (3.6), complete, 9/110 (8.1), incomplete	5/110 (4.5), complete-3; incomplete, 2 (1.8)	2/110 (1.8)—AVB I	7/110 (6.3)	-
Nordin [51]	-	-	1/110 (0.9)	8/110 (7.2)	2/110 (1.8)—AVB I 1/110 (0.9)—AVB II	2/110 (1.8)	-
Poormoghim [53]	-	-	1/58(1.7)	2/58 (3.4)	-	4/58 (6.8)	-
Radwan [54]	No	-	11/78 (14.1) (1, baseline; 10, FU)	1/78 (1.2)	21/78 (26.9)—AVB I (9 (11.5), baseline; 12 (15.3), FU) 1/78 (1.2)—AVB II 1/78 (1.2)—AVB III	-	-
Roberts [56]	-	-	-	-	4/50 (8.0)—AVB I 1/50 (2.0)—AVB III	8/50 (16.0)	-
Sano [58]	-	-	4/40 (10.0)	1/40 (2.5)	3/40 (7.5)—AVB I	3/40 (7.5)	1/40 (2.5)
Sergiacomi [60]	-	-	3/17 (17.6)	-	-	2/17 (11.7)	-
Tzelepis [61]	No	-	2/36 (5.5)	1/36 (2.7)	-	-	-
Wranicz [64]	-	-	-	1/22 (4.5)	-	1/22 (4.5)	-

RBBB, right bundle branch block; LBBB, left bundle branch block; AVB, atrioventricular-block; LAFB, left anterior fascicular block; LPFB, left posterior fascicular block; N, total number of patients; n, number of patients with conduction disturbances; %, percentage of patients with conduction disturbances.
